# Evolution of floral traits and mating systems under drought: a range-wide study of *Mimulus cardinalis*

**DOI:** 10.1093/aobpla/plaf062

**Published:** 2025-11-28

**Authors:** Olivia Wilborn-Pilotte, Emily Cook, Katelin Kutella, Seema Nayan Sheth, Jeff Diez

**Affiliations:** Institute of Ecology and Evolution, Department of Biology, University of Oregon, 272 Onyx Bridge, Eugene, OR 97401, United States; Institute of Ecology and Evolution, Department of Biology, University of Oregon, 272 Onyx Bridge, Eugene, OR 97401, United States; Institute of Ecology and Evolution, Department of Biology, University of Oregon, 272 Onyx Bridge, Eugene, OR 97401, United States; Department of Plant and Microbial Biology, North Carolina State University, Campus Box 7612, Raleigh, NC 27695, United States; Institute of Ecology and Evolution, Department of Biology, University of Oregon, 272 Onyx Bridge, Eugene, OR 97401, United States; Natural History and Conservation

**Keywords:** floral evolution, drought, *Erythranthe cardinalis*, hummingbird pollination, selfing

## Abstract

Climate change is intensifying droughts across the globe, challenging species to adapt to novel conditions. While plant physiological and phenological responses to drought are well-documented, less is known about how water scarcity affects the evolution of selfing across species ranges. According to the selfing syndrome hypothesis, in environments where selfing confers a fitness advantage, selection should favour floral traits associated with increased selfing relative to outcrossing. We used a field experiment near the northern range edge of the scarlet monkeyflower (*Mimulus cardinalis*) to test this hypothesis both spatially (among leading-edge, central, and trailing-edge populations), and temporally (between cohorts separated by a period of historic drought). Although populations from different range positions showed genetic differentiation in some floral traits, these differences did not consistently support predictions of the selfing syndrome hypothesis. Contrary to the predictions of reduced investment in floral rewards and increased selfing ability at range edges, the sugar content of nectar was greater and autogamous seed set was smaller in leading-edge than central populations, herkogamy tended to be greater in trailing-edge populations relative to leading-edge and central ones, and nectar volume did not vary predictably among regions. There was no support for the evolution of selfing syndrome from the predrought ancestors to the postdrought descendants. Instead, in leading-edge populations, descendants evolved greater sugar content relative to ancestors, and there were no other differences between ancestors and descendants in any other trait or region. Overall, these findings suggest that mating system evolution in *M. cardinalis* likely reflects a complex interplay of regional factors including range position, historical adaptation, and local environmental variability, rather than simple stress-induced shifts towards selfing.

## Teaser

Climate change is intensifying droughts globally, challenging species to adapt to novel conditions. While plant physiological and phenological responses to drought are well-documented, less is known about how water scarcity affects selfing evolution across species ranges. Drought stress may favor traits promoting self-fertilization over outcrossing. We tested this using scarlet monkeyflower populations across their range, comparing traits spatially (among leading-edge, central, and trailing-edge populations) and temporally (before and after historic drought). Contrary to predictions, we found little support for evolution toward selfing after extreme drought or at range edges. Instead, poleward populations evolved greater nectar sugar content, promoting outcrossing. Our findings suggest mating system evolution reflects complex regional factors rather than simple stress-induced shifts toward selfing.

## Introduction

Within species, populations have evolved adaptations to climate across both space and time. Evidence suggests that populations can adapt to changing conditions on contemporary timescales (Hairston et al. 2005, Franks et al. 2007, Anstett et al. 2021), which is particularly important for understanding how species may respond to the most extreme environmental changes such as megadroughts and heat waves. These rapid adaptations manifest in plants' morphology, physiology, and phenology, and may enable populations to persist despite shifting environmental pressures throughout their geographic range and over time (Bell 2017).

Mating system characteristics strongly influence how plants adapt to environmental change across spatial and temporal gradients. Floral traits evolve over time in response to both biotic factors (such as pollinator abundance) and abiotic factors, including water availability, temperature, and light (Lankinen et al. 2017, Caruso et al. 2019, Day Briggs and Anderson 2024). Plants with a mixed mating system produce seed via a combination of self-pollination and outcrossing, and fundamental pollination strategies evolve in response to varying ecological conditions across landscapes and through climatic shifts (Bontrager and Angert 2016, Bishop et al. 2023). The two distinct reproductive approaches, outcrossing and selfing, present evolutionary trade-offs: outcrossing demands more energy for developing pollinator-attractive traits but enhances genetic diversity, while selfing conserves energy and provides reproductive assurance in the absence of mates or pollinators but results in more genetically homogeneous populations that could suffer from inbreeding depression (Cheptou 2019).

According to the selfing syndrome hypothesis, selection should lead to evolutionary responses in floral traits associated with increased selfing ability in environments where selfing confers a fitness advantage (Darwin 1876, Ornduff 1969, Cutter 2019, Cisternas-Fuentes et al. 2022). The agents of selection could be abiotic (e.g. extreme drought or heat), biotic (e.g. low pollinator abundance), or a combination (e.g. severe drought reduces pollinator abundance). The selfing syndrome hypothesis predicts that in environments with reduced selection for floral rewards, nectar production and sugar content may decrease as plants invest less energy in producing these rewards in environments that favour selfing (Sicard and Lenhard 2011, Liao et al. 2022). In addition, herkogamy, the spatial separation between anthers and stigma within a flower, should decrease since selfing is more likely if anthers and stigma are closer together (Fenster and Ritland 1994, Karron et al. 1997).

For selfing to persist in the long term, the fitness benefits gained through reproductive assurance and energy savings must outweigh the costs associated with reduced genetic variation and inbreeding depression, which can cause a reduction in fitness due to increased homozygosity exposing recessive deleterious alleles, and potentially from the loss of heterozygote advantage (Byers and Waller 1999, Charlesworth and Willis 2009). Inbreeding can also increase the expression of deleterious alleles, allowing them to be purged by selection, particularly in small populations where genetic drift may also play a role, although this outcome is not guaranteed (Byers and Waller 1999, Winn et al. 2011). For example, some range-expanding species have shown evidence of purging, facilitated by repeated bottlenecks during colonization (Pujol et al. 2009). However, inbreeding depression is often only partially reduced, and residual fitness costs can persist even in predominantly selfing populations (Byers and Waller 1999, Winn et al. 2011). Thus, the evolutionary trajectory of selfing lineages reflects a balance between the potential for purging and the enduring costs of inbreeding depression.

While pollinator availability is clearly a key agent of selection on floral traits, researchers have also examined how environmental conditions influence plant mating systems (Whitehead et al. 2018), revealing nuanced and sometimes contradictory patterns. A meta-analysis found that selection from abiotic factors on floral traits is as strong as pollinator-driven selection on average (Caruso et al. 2019). However, individual studies have yielded inconsistent results across species and experimental conditions, finding both increases and decreases in selfing rates in response to elevated temperatures. For example, in a controlled study of *Viola praemorsa*, plants grown in warming chambers with restricted rainfall and elevated temperatures showed significantly increased rates of selfing compared to those in ambient conditions, suggesting that selfing may serve as an adaptive mechanism ensuring reproduction under environmental stress (Jones et al. 2013). Conversely, when researchers applied an experimental heatwave to *Vicia faba*, they discovered the opposite pattern, with plants under warming treatments exhibiting higher rates of outcrossing compared to controls (Bishop et al. 2016).

Given the potential effects of drought and temperature stress on mating system evolution, floral traits should evolve in response to spatial climatic gradients across species’ geographic ranges. Because populations at the edges of species’ ranges are often more isolated, smaller in size, and occupy more marginal climates relative to core populations, they could evolve increased selfing rates and shifts in floral traits that increase selfing (Pannell et al. 2015). At leading range edges (e.g. poleward or high elevation), where populations are expanding into newly suitable climates, selfing could provide reproductive assurance in the absence of sufficient mates or pollinators (Eckert et al. 2010, Hargreaves and Eckert 2014). Similarly, at the trailing edges of species’ ranges (e.g. towards equator or low elevation), where populations are contracting from newly unsuitable climates, reductions in mate and/or pollinator availability could select for increased selfing (Eckert et al. 2010, Levin 2012).

As climate change exacerbates heat and drought stress, extreme climate events such as major droughts could also lead to evolutionary shifts in floral traits towards selfing (Day Briggs and Anderson 2024). For instance, drought could either limit mate and/or pollinator availability (Day Briggs and Anderson 2024), or limit the amount of water that plants can allocate to floral rewards for pollinators (Kuppler and Kotowska 2021), potentially leading to reduced nectar production. The effects of drought on nectar sugar content are less straightforward. If limited water availability results in smaller nectar volume within flowers, then the sugar content of that nectar could increase (Kuppler and Kotowska 2021). For hummingbird-pollinated plants, the focus of our study, flowers typically contain more dilute nectar relative to bee-pollinated plants (Liao et al. 2022), but behavioural observations suggest that hummingbirds prefer more concentrated nectar (Roberts 1996). However, if limited water availability favours selfing due to the energy savings and reproductive assurance, then plants may invest less in sugar production as a floral reward for pollinators. Either way, spatial or temporal changes in water availability could potentially alter nectar sugar content in ways that impact the likelihood of selfing. There is also evidence that drought can lead to reduced herkogamy (Opedal et al. 2016). All of these floral traits could respond plastically to environmental variation (Kay and Picklum 2013), however there is growing evidence for evolutionary shifts in floral traits in response to these types of spatial and temporal climatic variation (Opedal et al. 2017, Cheptou et al. 2022, Bishop et al. 2023).

In this study we evaluate the selfing syndrome hypothesis across the range of *Mimulus cardinalis* (syn. *Erythranthe cardinalis)*, a self-compatible, hummingbird-pollinated species with a geographic range that spans a broad latitudinal and climatic gradient. To do so, we studied plants from one common garden that was part of a broader range-wide resurrection study of quantitative genetics and evolutionary responses in physiological and phenological traits in response to a record-setting drought in western North America. We focused on two floral traits associated with pollinator attraction (nectar volume and nectar sugar content) and two traits associated with selfing ability (herkogamy and seed set in the absence of pollinators). According to the selfing syndrome hypothesis, we predicted that populations experiencing greater environmental stress, either spatially at the edges of the species’ range or temporally after a historic drought event, would evolve reproductive traits associated with increased selfing (e.g. smaller nectar volume, sugar content, and herkogamy and greater autogamous seed set) as a potential means to conserve resources and/or ensure reproduction under challenging conditions. This expectation aligns with theoretical predictions that selfing provides reproductive assurance when ecological conditions limit outcrossing opportunities (Baker 1955, Barrett 2003). This study provides the first step to understanding how the evolution of selfing contributes to population persistence across environmental gradients in both space and time, potentially serving as a mechanism of evolutionary rescue as climate conditions continue to change across a species' range (Cheptou et al. 2022).

## Methods

### Study system

**Figure 1.**
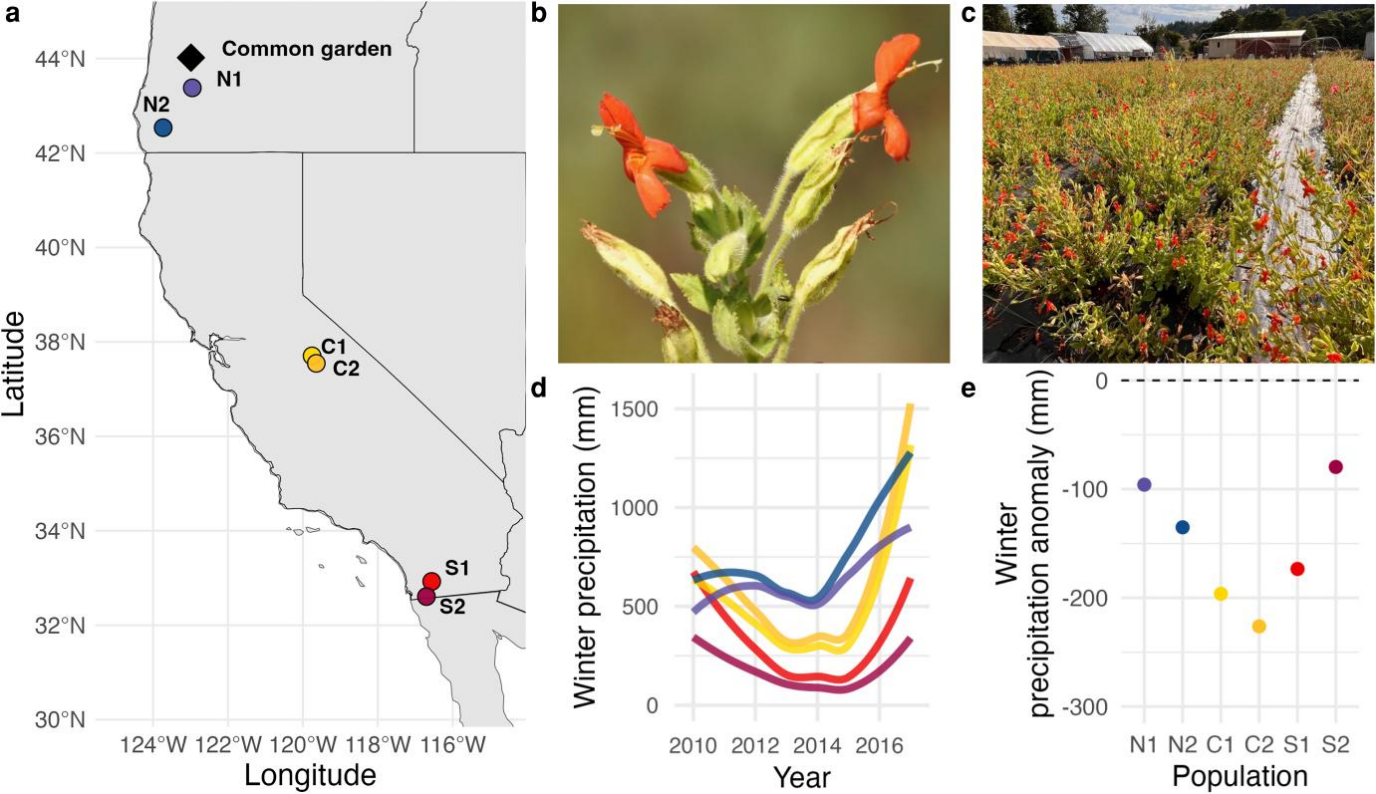
(a) Map of focal source populations and the location of the common garden experiment (red diamond). (b) *Mimulus cardinalis* plant showing open stigma and anthers. (c) Common garden in Eugene, OR. (d) Mean yearly winter precipitation of focal populations during drought. (e) Precipitation anomalies for each focal population calculated by subtracting mean winter precipitation between 2012 and 2015 by the historical 30-year averages (1981–2010). Climate data for each focal population are derived from climateNA v. 7.30 (Wang et al. 2016).

*Mimulus cardinalis* (Phrymaceae) is a perennial herb that grows in riparian habitats, particularly in rocky streambeds and seeps, generally found below 2400 m in elevation. Its range extends from central Oregon, USA into northern Baja California, Mexico, and from the California coast inland towards Nevada (Fraga 2018). As a hummingbird-pollinated species with a mixed mating system, *M. cardinalis* can both outcross and self-pollinate (Nelson et al. 2021), and geitonogamy, whereby self-pollination occurs between different flowers on the same individual plant, is possible (Sheth, *pers. obs.*). The species belongs to the *Mimulus* complex, a model system for studying evolution due to its wide array of phenotypic, ecological, and genetic variation across species and populations (Fishman et al. 2001, Wu et al. 2008). *Mimulus cardinalis* has been the focus of studies examining demographic (Sheth and Angert 2018) and physiological adaptation in response to climate change (Sheth and Angert 2016, Muir and Angert 2017, Muir et al. 2022), particularly because its dependence on water availability makes it ideal for studying trait evolution in response to drought (Nelson et al. 2021). Specifically, *M. cardinalis* inhabits areas with flowing water for at least some part of the growing season (Sheth, *pers. obs.*). Furthermore, as a species that spans a large geographic and climatic range (Fig. 1), *M. cardinalis* experiences varying selection pressures across its distribution, making it an excellent system for studying evolution more broadly (Kooyers et al. 2025).

### Experimental design

This study takes advantage of a common garden that is part of a larger resurrection experiment focused on quantifying quantitative genetic parameters and evolutionary responses to a historic period of drought across the range of *M. cardinalis* (Fig. 1; Diffenbaugh et al. 2015). For this larger study, seeds were collected from six populations of *M. cardinalis* (two northern-edge, two central, and two southern-edge; Fig. 1) in 2010 (‘predrought’ ancestors) and 2017 (‘postdrought’ descendants), before and after a period of historic drought, respectively (Sheth and Angert 2016, Vtipil and Sheth 2020, Wooliver et al. 2020, Sheth et al. 2026). Locality information for each population is reported in Sheth and Angert (2016). To control for maternal and seed storage effects, plants were crossed for one generation following a nested paternal half-sibling design to allow for the estimation of quantitative genetic parameters (Sheth et al. 2026). After 5 weeks of greenhouse growth, we transplanted seedlings into three common gardens across the range in a randomized block design. Due to logistical reasons that prevented collection of floral trait data in the central and southern gardens, this study focuses on data from the northernmost garden in Eugene, Oregon (Friends of Buford Park and Mount Pisgah Native Plant Nursery). The garden had 10 blocks, with all six populations and cohorts represented in each block, for a total of 5468 individuals. For this study of floral traits, we randomly selected 180 plants (30 from each region and cohort) from different maternal families to use as study plants.

To quantify differentiation in traits that might be important for mating system across space (among populations) and time (between cohorts), we used a pollination exclusion experiment to compare floral rewards, morphology, and seed set between populations. We enclosed two buds per plant in mesh bags to prevent pollinator access before they opened. For one bagged flower per plant, we measured three traits associated with mating system. First, we measured nectar volume as an indicator of the amount of reward that the flower provides to pollinators, such that flowers producing less nectar may exhibit a greater degree of selfing than flowers producing more nectar (Sicard and Lenhard 2011). We measured nectar volume in micro-litres using a 30 μl microcapillary tube inserted into the nectary. Volume was calculated from the height of the nectar column, measured with digital callipers (Kearns and Inouye 1993). Second, we measured nectar sugar content as a metric of quality of the floral reward, with greater nectar sugar content generally associated with outcrossing (Bartoš et al. 2020). We used a refractometer (model SR0017-ATC from manufacturer Xindacheng) to measure nectar sugar content. Samples were diluted with 50 μl of deionized water, and the undiluted sugar content (measured in degrees Brix, Brix°, where 1°Brix equals 1 g of sucrose in 100 g of solution) was calculated using the formula:


$$\begin{array}{rl} \mathrm{dilution}\;\;\mathrm{factor} & =\mathrm{total}\;\;\mathrm{sample}\;\;\mathrm{volume}\;\;(\mathrm{nectar}+\mathrm{water})/\\ & \;\mathrm{nectar}\;\mathrm{volume} \end{array}$$

$$\mathrm{undiluted}\;\mathrm{Bri}x^{o}=\mathrm{dilution}\;\mathrm{factor}\times \mathrm{diluted}\;\mathrm{Bri}x^{o}$$

Third, we measured herkogamy, the spatial separation of stigma and anthers in a flower, which should influence the probability of selfing, with shorter absolute distance between anthers and stigma associated with greater selfing rates (Chang and Rausher 1999, Opedal 2018). To estimate herkogamy, we used digital callipers to measure the distance between the uppermost another pair and stigma. To evaluate the potential efficacy of autonomous selfing, we collected fruits resulting from the second bagged flower on each plant to assess autonomous seed set as plants senesced. Due to the high seed count per fruit and multiple fruits per plant, we estimated the total seed set per fruit based on mass (Angert 2006). On a subset of fruits, we counted the number of seeds using photographs (Nikon D750 camera) of seeds on a white background. Images were analysed with ImageJ to isolate the seeds, and seeds were then weighed on an analytical balance. From this subset, seed number and seed mass were used to build a relationship to predict seed number for the remaining fruits based on their seed mass using the predict() function in R.

### Analysis

We analysed differences in floral traits across populations and cohorts using linear mixed-effects models implemented in the lme4 package (Bates et al. 2015) in R (R Core Team 2025) and lmerTest (Kuznetsova et al. 2017) for estimating significance of fixed effects. For each floral trait (nectar sugar concentration, nectar volume, herkogamy), and seed set, we constructed separate models of the form:


$$\begin{array}{rl} \mathrm{floral}\;\mathrm{trait} & ∼\mathrm{Region}+\mathrm{Cohort}+\mathrm{Region}\times \mathrm{Cohort}\\ & \;+(1|\mathrm{block}) \end{array}$$

where Region (north, central, south) and Cohort (predrought 2010 ancestors, postdrought 2017 descendants) were fixed effects, and block was included as a random effect to account for potential environmental heterogeneity within the common garden. We conducted *post hoc* tests using the emmeans package (Lenth and Piaskowski 2025) to evaluate pairwise differences among regions and cohorts. Tukey's HSD adjustment was applied to control for multiple comparisons. We obtained estimated marginal means and contrasts for (i) region averaged across cohorts, (ii) cohort averaged across regions, and (iii) effects of cohort within each region.

To investigate whether floral traits varied independently or covaried, we calculated Pearson's correlation coefficients between all pairs of response variables. Statistical significance was assessed at *α* = 0.05 for all analyses. Model assumptions of normality and homoscedasticity were verified through visual inspection of residual plots.

## Results

### Correlations among variables

Pearson correlation coefficients revealed a weakly significant negative relationship between nectar volume and nectar sugar, and a weakly negative correlation between nectar sugar and herkogamy; all other relationships between response variables were not significant (Supplementary Figure S1).

### Regional and temporal effects on floral traits

#### Nectar traits: sugar concentration and volume

Nectar sugar concentration varied significantly among regions, with a marginally significant interaction between region and cohort but no significant main effect of cohort (Table 1). *Post hoc* comparisons revealed that populations from the central region had significantly smaller nectar sugar concentrations than those from the northern region, while differences between southern and central regions and southern and northern regions were not significant (Supplementary Table S1).

**Table 1.** ANOVA table for the four response variables.

Variable	Effect	Sum sq	Mean sq	NumDF	DenDF	*F* value	Pr(>*F*)
Nectar sugar content (°Bx)	Region	316.2	158.1	2	168.5	4.6	.0109*
Nectar sugar content (°Bx)	Year	75.2	75.2	1	162.0	2.2	.1392
Nectar sugar content (°Bx)	Region:year	185.0	92.5	2	165.7	2.7	.0691
Herkogamy [anther stigma distance (mm)]	Region	9.6	4.8	2	170.0	3.1	.0472*
Herkogamy [anther stigma distance (mm)]	Year	0.1	0.1	1	170.0	0.0	.8335
Herkogamy [anther stigma distance (mm)]	Region:year	5.3	2.7	2	170.0	1.7	.1794
Nectar volume (μl)	Region	162.0	81.0	2	171.0	2.4	.0904
Nectar volume (μl)	Year	2.0	2.0	1	163.6	0.1	.8080
Nectar volume (μl)	Region:year	6.2	3.1	2	168.3	0.1	.9112
Predicted seed number (log)	Region	9.3	4.7	2	165.0	5.8	.0039**
Predicted seed number (log)	Year	0.0	0.0	1	165.0	0.0	.8362
Predicted seed number (log)	Region:year	3.3	1.7	2	165.0	2.1	.1312

**P < .01, *P < .05, *P* < .1.

**Figure 2.**
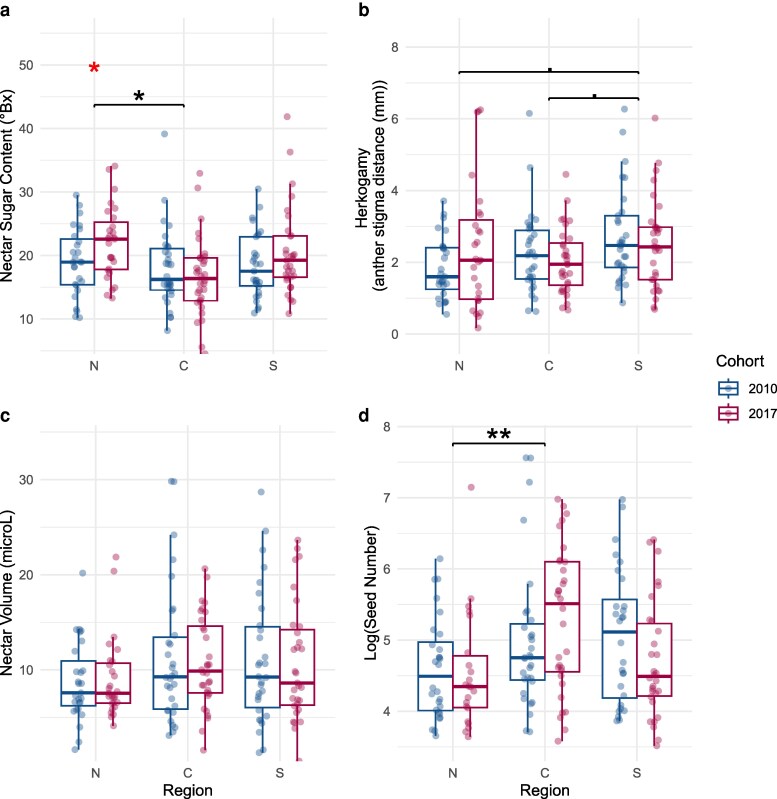
Values of (a) nectar sugar, (b) herkogamy (absolute value of the anther stigma distance), (c) nectar volume, and (d) seed number (log scale) in North, Central, and South regions before (2010) and after (2017) drought. Significance of difference among regions is shown with bars and black asterisks; significance of cohort effects within regions are shown with a red asterisk. In all cases: **P < 0.01, *P < .05, *P* < 0.1.

The most notable difference between cohorts occurred in plants from the northern region, where descendants produced significantly greater nectar sugar concentrations (22.23 ± 1.60%) compared to ancestors (19.04 ± 1.21%) (Supplementary Table S1), suggesting increased investment in pollinator rewards following the historic drought event in northern populations. However, central and southern populations showed no significant changes in nectar sugar concentration between predrought ancestors and postdrought descendants (Fig. 2, Supplementary Table S1).

In contrast to other floral traits, nectar volume did not vary significantly across regions or between cohorts; nor was there a significant interaction between region and cohort (Table 1, Fig. 2).

#### Herkogamy

Herkogamy, a key morphological trait influencing the capacity for selfing, varied significantly by region (Table 1; Fig. 2), with the greatest herkogamy in the southern populations and smallest herkogamy in the northern populations. *Post hoc* comparisons showed marginally significant differences between plants from the southern and central regions (*P* = .094) and between plants from the southern and northern regions (*P* = .082), while plants from the central and northern regions did not differ significantly (Supplementary Table S1). There was not a significant difference between the cohorts nor a significant interaction between region and cohort on herkogamy (Table 1).

#### Seed set

Seed set varied significantly among regions, with plants from the central region having the highest seed set and the northern populations having the lowest (Table 1). There were not significant effects of cohort nor region-cohort interactions (Table 1) and within-region temporal comparisons showed no significant changes between cohorts in any region (Supplementary Table S1). *Post hoc* comparisons confirmed that the central region had significantly greater seed set than the northern region, while differences between the southern and central regions and southern and northern regions were not significant (Supplementary Table S1).

## Discussion

We used a field experiment near the northern range edge of the scarlet monkeyflower to test the selfing syndrome hypothesis both spatially (among leading-edge, central, and trailing-edge populations), and temporally (between cohorts separated by a period of historic drought). This hypothesis predicts that populations experiencing stressful environments, such as those at range edges or following extreme climatic events, may evolve floral traits associated with increased selfing relative to outcrossing, including smaller nectar production, nectar sugar content, and herkogamy, and greater autogamous seed set. Although populations from different range positions showed genetic differentiation in some floral traits, these differences did not consistently support predictions of the selfing syndrome hypothesis. Contrary to the predictions of reduced investment in floral rewards and increased selfing ability at range edges, the autogamous seed set was smaller in leading-edge than central populations, herkogamy tended to be greater in trailing-edge populations relative to leading-edge and central ones, and nectar volume did not vary predictably among regions. However, sugar content of nectar was greater in the northern region compared to the central region. There was no support for the evolution of selfing syndrome from the predrought ancestors to the postdrought descendants. Instead, in leading-edge populations, descendants evolved greater sugar content relative to ancestors, and there were no other differences between ancestors and descendants in any other trait or region. Overall, these findings suggest that mating system evolution in *M. cardinalis* reflects a complex interplay of regional factors including range position, historical adaptation, and local environmental variability, rather than simple stress-induced shifts towards selfing. Below, we discuss these floral trait patterns in relation to range limits and climate change.

### Evolution of floral traits across space

Among populations from different regions, there were distinct patterns of floral trait variation that challenge simple predictions of the selfing syndrome hypothesis. Contrary to our expectations, southern, trailing-edge populations tended to exhibit greater herkogamy (Fig. 2) compared to central and northern, leading-edge populations. This finding is consistent with a meta-analysis that found that outcrossing rates decreased with increasing latitude, although this relationship was predominately driven by variation in life form (i.e. a greater frequency of longer-lived plants in the tropics relative to temperate areas) rather than pollination environment (Moeller et al. 2017). The abundance of pollinators (and therefore more reliable outcrossing) could also help explain variation in herkogamy, but we observed hummingbirds at all sites and lack data on their abundances. Also contrary to the prediction that selfing would be more prevalent at range margins, central populations demonstrated greater seed set under obligate selfing conditions (bagged flowers) compared to northern populations. Overall, these results suggests that other site-level factors may influence the evolution of selfing, and that pollinator and mate availability may not always vary predictably across climatic gradients or species’ geographic ranges.

### Evolution of floral traits over time

Results also showed limited evolution of floral traits from predrought ancestors to postdrought descendants. In plants from the leading range edge, we observed greater nectar sugar concentrations in postdrought descendants compared to predrought ancestors. Indicative of the evolution of increased investment in traits associated with pollinator rewards, this result is consistent with a resurrection study of the common morning glory (*Ipomopsis purpurea*), an annual weed, which documented an increase in nectar sugar content from ancestors to descendants, especially in high latitude populations (Bishop et al. 2023). This increase in nectar sugar may have particular significance at the expanding range edge for *M. cardinalis*, as outcrossing can be especially advantageous by promoting genetic variability in colonizing populations, potentially facilitating adaptation to novel conditions (Sexton et al. 2011). However, nectar volume did not evolve from ancestors to descendants, perhaps reflecting the high plasticity of nectar production to current environmental conditions, which may slow evolutionary responses (Ghalambor et al. 2015, Parachnowitsch et al. 2019). A meta-analysis of studies investigating the effect of a drought manipulation on floral traits found a reduction in nectar volume in drought treatments, especially in indoor studies (Kuppler and Kotowska 2021), suggesting that even in the absence of pollinators, nectar volume can plastically respond to water availability.

The lack of evolutionary shifts in herkogamy between ancestors and descendants could be due to the potential for geitonogamy, whereby self-pollination occurs when hummingbirds or other animal pollinators transfer pollen from one flower to another on the same plant, relaxing selection for reduced herkogamy following drought conditions. We have indeed observed hummingbirds visit multiple flowers on the same plants in all three common gardens in the broader experiment (Sheth, *pers. obs*.). Unlike a resurrection study of the field pansy (*Viola arvensis*), an annual plant with a mixed mating system, we did not find evidence for the evolution of increased autogamous seed set from ancestors to descendants (Cheptou et al. 2022). However, the study of *Viola arvensis* was conducted over a longer time period (with ancestors and descendants separated by two decades) and in the context of rising temperatures and pollinator declines in agrosystems. This highlights the value of future work investigating changes in pollinator availability and over longer time periods.

It's possible that the observed lack of evolutionary change in most floral traits from ancestors to descendants reflects adaptational lags in these source populations. In the broader study, across the northern, central, and southern common gardens, we have observed patterns of local maladaptation in the northern and central populations. In particular, in the first growing season (2023), southern populations had greater fitness (estimated based on the total number of reproductive structures on each plant and maximum stem height at the end of the growing season) than northern and central populations in the northern and central gardens, irrespective of cohort (Sheth et al. 2026).

### Caveats

Several caveats should be considered when interpreting our results. First, our fecundity measurements were limited to obligately selfing (bagged) flowers, leaving open questions about relative reproductive success under natural pollination conditions. Measuring reproductive success under both selfing and outcrossing conditions would allow deeper exploration of these relationships. Further, using genetic data to directly quantify selfing rates and variation in mating systems would allow for a more robust test of how mating system varies across species ranges that span broad environmental gradients. Second, while our methodology controlled for pollinator visitation through bagging and standardized sampling times, the inherently high variability in nectar production among individual plants and through the season may have obscured any evolutionary patterns. A valuable follow-up study would be to sample floral traits from multiple flowers and at multiple time points throughout the growing season to account for temporal and intra-individual variation in these traits. Third, because our study was conducted in a single common garden, it does not reveal whether floral traits exhibit phenotypic plasticity in response to environmental variation. Ideally, trait expression and its relationship to fitness would be assessed across multiple common gardens spanning the species’ range, or under experimental climate change scenarios, to quantify the extent of plasticity present in these populations (Day Briggs and Anderson 2024). Finally, while we examined several of the key floral traits thought to influence selfing in bird-pollinated plants, there are additional floral traits such as corolla size, pollen count, pollen viability, and petal pigmentation that could play a role in evolutionary shifts in mating system (Sicard and Lenhard 2011). For instance, a recent study of latitudinal variation in floral traits of *M. cardinalis* found key differences in the biochemistry of floral scent and pigmentation (Neequaye et al. 2025).

### Conclusions

This study examines floral trait evolution across both space and time using a resurrection approach. We found limited evolutionary shifts in floral traits between ancestral and descendant cohorts from before and after an extreme drought event, likely associated with adaptational lags in many of the source populations. Despite the evolution of spatial differences in some floral traits, the direction of change did not support the hypothesis that populations from range edges would exhibit floral traits that enhance selfing ability (Pannell et al. 2015). Despite theoretical expectations of increased selfing ability, especially towards leading range edges, these findings suggest that leading and trailing range edges can behave differently, and that floral traits associated with selfing may not always evolve in concert across species’ geographic ranges. Future research combining trait measurements with population surveys and demographic modelling would help link trait variation to fitness outcomes in natural populations, ultimately allowing us to better predict whether and how selfing might facilitate evolutionary rescue under changing environmental conditions (Degottex-Féry and Cheptou 2023). Further, common garden studies that explicitly investigate both abiotic and biotic agents of selection on floral traits will help untangle the complex, interacting factors shaping floral evolution in nature (Day Briggs and Anderson 2024).

## Supplementary Material

plaf062_Supplementary_Data

## Data Availability

Dataset has been submitted to Dryad (9/15/2025). http://datadryad.org/share/LINK_NOT_FOR_PUBLICATION/ZcwOijwHS8tpRJ03nVVQqI8oAbHNyCjfz96OrZgVOGE
