# Supplementary material for: Evolution of floral traits and mating systems under drought: a range-wide study of Mimulus cardinalis
Source: AoB Plants. 2025 Nov 28;17(6):plaf062. doi: 10.1093/aobpla/plaf062 (PMC12662168; doi:10.1093/aobpla/plaf062)
Supplement: plaf062_Supplementary_Data [file plaf062_supplementary_data.pdf]

Supporting Information

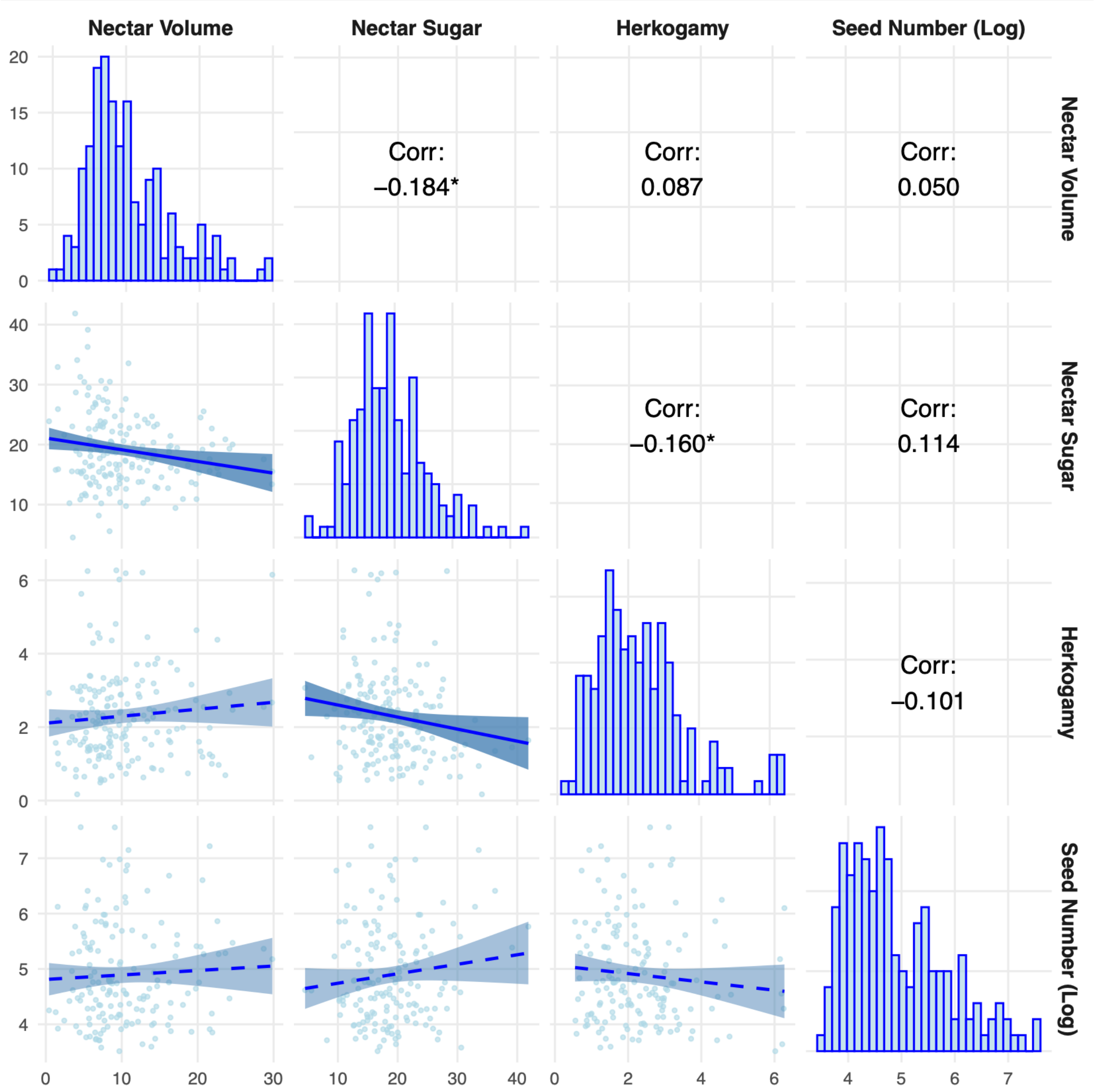

Figure S1: Correlations among variables of interest, with Pearson correlation coefficients (upper right triangle). Lines (in bottom left triangle) are linear regression plots in ggplot, with solid lines for significant correlation coefficients and dashed lines for non-significant ($p > 0.05$) correlations. Units are: Nectar Volume (μL); Nectar Sugar Content (°Bx); Herkogamy (absolute value of distance between anther and stigma); Seed Number (log number of seeds).

Table S1. Table of post-hoc contrasts from regression models of the form: *variable ~ region x cohort + (1 | block)*

Variable	Type	Contrast	Est.	SE	df	t	p	
Nectar Sugar Content (°Bx)	Region	S - C	2.22	1.09	169.41	2.04	0.105	
Nectar Sugar Content (°Bx)	Region	S - N	-1.04	1.11	167.31	-0.94	0.618	
Nectar Sugar Content (°Bx)	Region	C - N	-3.26	1.11	168.98	-2.94	0.010	*
Nectar Sugar Content (°Bx)	Cohort within	Year2010 - Year2017 | S	-2.31	1.52	164.58	-1.52	0.131	
Nectar Sugar Content (°Bx)	Cohort within	Year2010 - Year2017 | C	1.56	1.51	166.97	1.04	0.301	
Nectar Sugar Content (°Bx)	Cohort within	Year2010 - Year2017 | N	-3.20	1.60	164.48	-2.00	0.048	*
Herkogamy (Anther Stigma	Region	S - C	0.48	0.23	168.55	2.10	0.094	.
Herkogamy (Anther Stigma	Region	S - N	0.51	0.24	169.84	2.15	0.082	.
Herkogamy (Anther Stigma	Region	C - N	0.03	0.23	169.99	0.12	0.992	
Herkogamy (Anther Stigma	Cohort within	Year2010 - Year2017 | S	0.21	0.32	166.62	0.64	0.525	
Herkogamy (Anther Stigma	Cohort within	Year2010 - Year2017 | C	0.22	0.32	169.79	0.69	0.491	
Herkogamy (Anther Stigma	Cohort within	Year2010 - Year2017 | N	-0.54	0.34	167.33	-1.60	0.112	
Nectar Volume (µL)	Region	S - C	-0.11	1.06	170.65	-0.11	0.994	
Nectar Volume (µL)	Region	S - N	2.03	1.09	170.51	1.86	0.155	
Nectar Volume (µL)	Region	C - N	2.14	1.09	170.94	1.97	0.124	
Nectar Volume (µL)	Cohort within	Year2010 - Year2017 | S	0.28	1.49	167.37	0.19	0.851	
Nectar Volume (µL)	Cohort within	Year2010 - Year2017 | C	0.64	1.48	170.15	0.43	0.666	

Variable	Type	Contrast	Est.	SE	df	t	p	
Nectar Volume (µL)	Cohort within	Year2010 - Year2017 | N	-0.29	1.58	167.41	-0.18	0.857	
Predicted Seed Number (log)	Region	S - C	-0.26	0.17	163.82	-1.59	0.254	
Predicted Seed Number (log)	Region	S - N	0.32	0.18	163.22	1.80	0.173	
Predicted Seed Number (log)	Region	C - N	0.58	0.17	164.88	3.35	0.003	**
Predicted Seed Number (log)	Cohort within	Year2010 - Year2017 | S	0.35	0.24	164.04	1.47	0.143	
Predicted Seed Number (log)	Cohort within	Year2010 - Year2017 | C	-0.31	0.23	163.63	-1.35	0.178	
Predicted Seed Number (log)	Cohort within	Year2010 - Year2017 | N	0.05	0.26	164.00	0.18	0.856	

*** p < 0.001, ** p < 0.01, * p < 0.05, . p < 0.1

Table S2. Sample sizes (numbers of plants) from unique sires, dams and sire-dams (unique crosses) in the study for each region and year (cohort).

Region	Year	Unique Sires	Unique Dams	Unique Crosses	Total N
S	2010	26	30	30	32
S	2017	23	32	32	36
C	2010	21	30	30	33
C	2017	23	31	31	35
N	2010	15	28	28	30
N	2017	23	26	26	29
